# Activated CD4^+ ^T cells enhance radiation effect through the cooperation of interferon-γ and TNF-α

**DOI:** 10.1186/1471-2407-10-60

**Published:** 2010-02-23

**Authors:** Yixiang Wang, Soroosh Radfar, Hung T Khong

**Affiliations:** 1 Mitchell Cancer Institute, University of South Alabama, Mobile, Alabama 36604-1405, USA; 2 Research Laboratory of Oral and Maxillofacial Surgery, Peking University School and Hospital of Stomatology, 22 Zhongguancun Nandajie, Haidian District, Beijing 100081, PR China; 3 Yale University School of Medicine, Department of Internal Medicine, Hematology Section, 333 Cedar Street, P.O. Box 208021, New Haven, Connecticut 06520-8021, USA

## Abstract

**Background:**

Approaches that enhance radiation effect may lead to improved clinical outcome and decrease toxicity. Here we investigated whether activated CD4+ T cells (aCD4) can serve as an effective radiosensitizer.

**Methods:**

CD4+ T cells were activated with anti-CD3 and anti-CD28 mAbs. Hela cells were presensitized with aCD4 or conditioned supernatant (aCD4S) or recombinant cytokines for 2 days, followed γ-irradiation. The treated cells were cultured for an additional 2 to 5 days for cell proliferation, cell cycle, and western blot assays. For confirmation, other cancer cell lines were also used.

**Results:**

Presensitization of tumor cells with aCD4 greatly increased tumor cell growth inhibition. Soluble factors secreted from activated CD4^+ ^T cells were primarily responsible for the observed effect. IFN-γ seemed to play a major role. TNF-α, though inactive by itself, significantly augmented the radiosensitizing activity of IFN-γ. aCD4S, but not IFN-γ or IFN-γ/TNF-α combination, was found to enhance the γ-irradiation-induced G2/M phase arrest. Bax expression was highly upregulated in Hela cells presensitized with aCD4S followed by γ-irradiation. The radio-sensitizing activity of aCD4 is not uniquely observed with Hela cell line, but also seen with other cancer cell lines of various histology.

**Conclusions:**

Our findings suggest possible molecular and cellular mechanisms that may help explain the radio-sensitization effect of activated lymphocytes, and may provide an improved strategy in the treatment of cancer with radiotherapy.

## Background

Activated T cells elaborate cytokines that are essential for an effective immune response [1]. These include IL-2, tumor-necrosis factor (TNF)-α, IL-21 (by activated CD4^+ ^T cells), granulocyte/macrophage-stimulating factor (GM-CSF), interferon (IFN)-γ, and other yet known cytokines. In addition to their immune modulatory activities, some cytokines also have direct effects on tumor cells and/or tumor vasculature. TNF-α is known to induce hemorrhagic necrosis in tumors [2,3]. In addition, TNF-α has also been shown to induce apoptotic and necrotic tumor cell death *in vitro *[4-7]. The hemorrhagic necrotic effect is much more potent when used in combination with chemotherapy in a rat sarcoma model [8]. However, in these studies TNF-α has no activity against tumor cell lines in *in vitro *assays, and exerts no synergy with chemotherapy or radiotherapy in this setting [8,9]. The synergistic effect of TNF-α combined with chemotherapy or radiotherapy is directed mainly against the tumor vasculature [10-12]. The interferons (IFNs) can exert direct effects on the proliferation, differentiation, and apoptosis of tumor cells [13-17]. IFN-γ has been shown to enhance the apoptotic effect of radiation on medulloblastoma cells [18]. However, responses to the IFNs are considerably variable, depending on tumor histologies, and resistance to the IFNs' effects has been reported in several tumor types [19-22].

In order to fully utilize the complete repertoire of cytokines secreted by activated lymphocytes in a simple yet effective manner, we hypothesize that nonspecifically-activated CD4^+ ^lymphocytes (aCD4) can pre-sensitize tumor cells to enhance the apoptotic effect of gamma radiation. The rationale for this strategy is the known ability of activated T cells to secret multiple different cytokines, which may regulate proliferation and apoptosis of tumor cells as described above. In addition, activated T cells may exert direct activity on tumor cells through apoptotic pathways such as the engagement of Fas ligand, which is highly expressed on activated T cells, and Fas receptor on tumor cells.

In this study, we have shown that pre-sensitization of tumor cells with aCD4 prior to γ-irradiation significantly enhanced cancer cell growth inhibition, and that soluble factors released by aCD4 were primarily responsible for the observed activity. IFN-γ was found to be the main cytokine that mediated this effect, and TNF-α, though inactive by itself, significantly augmented the radiosensitizing activity of IFN-γ. We also found that aCD4S, but not IFN-γ or IFN-γ/TNF-α combination, enhanced the γ-irradiation induced G2/M phase arrest. In addition, Bax expression was highly upregulated in Hela cells presensitized with aCD4S followed by γ-irradiation.

## Methods

### Cell Lines

Human cervical cancer cell lines, Hela and CaSki, glioma cell lines, LN229 and U373 and prostate cancer cell line DU145 were obtained from American Type culture Collection (ATCC; Manassas, VA). All cell lines were cultured in RPMI 1640 (Mediatech, VA) supplemented with 10% FBS (Fetal Bovine Serum) (Atlanta Biologicals, GA), 1 U/ml penicillin and 1 μg/ml streptomycin (Mediatech) (hereafter referred to as Complete Medium or CM). All cell lines were maintained in a humidified incubator at 37°C with 5% CO_2_.

### Isolation and activation of CD4+ T cells (aCD4)

Buffy coat was purchased from regional American Red Cross. Peripheral blood mononuclear cells (PBMC) were isolated from the Buffy Coat using Ficoll-Paque Plus (GE Healthcare, Uppsala, Sweden) and frozen prior to use. CD4+ T cells were isolated negatively from PBMC, using the "CD4^+ ^T cell isolation kit II" (Miltenyi Biotech, CA). CD4^+ ^T cells were activated immediately after isolation. The activation was performed over night, using 24-well plate coated with 5 μg/mL anti-CD3 (OKT3, Orthobiotech, NJ) and 1 μg/mL anti-CD28 (eBioscience, CA), with 300 IU/ml of IL-2. Activated CD4^+ ^T cells (aCD4) were harvested, washed and used immediately for *in vitro *experiments or labeled with a FITC-conjugated mouse anti human CD4^+ ^T cells (eBioscience) and analyzed for purity by flow cytometry (FACSVantage, Becton Dickinson, NJ). The activation of CD4^+ ^cells was determined by IFNγ secretion in an ELISA assay. All ELISA reagents, monoclonal antibodies and recombinant cytokines were purchased from Pierce (Rockford, IL). The ELISA was performed according to the manufacturer's instructions.

### Generation of conditioned supernatant from aCD4

aCD4 that were activated overnight as described above were collected, washed, and cultured in 24-well plate at 1 × 10^5^, 2 × 10^5^, or 4 × 10^5 ^cells/2 ml of CM for 48 hr to generate conditioned media (hereafter called 5 × 10^4^, 1 × 10^5 ^and 2 × 10^5 ^aCD4S, respectively). The conditioned media were passed through 0.2 μm filter and stored at -70°C until use.

### Cytokine array

The concentration of thirteen cytokines secreted by aCD4 was analyzed. CD4+ T cells were activated overnight as described above, collected, washed, and transferred to new wells and cultured for another 48 hrs. The supernatants were collected and sent to "LINCO Diagnostic Services, Inc." (St. Charles, MO) for analysis of 12 common Th1 and Th2 pro-inflammatory and anti-inflammatory cytokines, including IL-1β, IL-2, IL-4, IL-5, IL-6, IL-8, IL-10, IL-12p40, IL-12p70, IL-13, IFN-γ and TNF-α. The same supernatants were also used to determine GM-CSF concentrations, using the "human GM-CSF ELISA kit" (Pierce, Rockford, IL) according to the manufacturer's instructions.

### Cell growth inhibition assay

Tumor cells were plated at 4 × 10^4 ^cells/well with 0.8 ml CM in a 24-well plate overnight. The following day, 0.8 ml CM containing the indicated doses of aCD4 (direct cell-cell contact or via Transwell system) or CD4S was added. Forty eight hrs later, tumor cells were collected and gamma irradiated. In experiments where recombinant cytokines were used in place of aCD4 or aCD4S, various combinations of cytokines at similar concentrations to those detected in the aCD4S derived from 1 × 10^5 ^aCD4 cells/mL CM (1 × 10^5 ^aCD4S) were used to presensitize tumor cells. The irradiated tumor cells were plated at 5 × 10^3^/well, in triplicate, with 200 μl of 0-25% aCD4S in 96-well plate for 5 days. On day 5, cell viability was determined using "WST-1 cell proliferation reagent" (Roche, IN) according to the manufacturer's instructions. Finally, the absorbance was measured at 450 nm with an "Opsys MR plate-reader (Dynex Technologies Inc., VA), and a killing curve was established. A suboptimal radiation dose that decreased cell viability by approximately 15-40% was chosen to use in further experiments, to obtain a large window for clear observation of the effect, if any, of the experimental treatment. All experiments were performed in triplicate and repeated at least 2 times.

### Neutralization of IFN-γ in aCD4S

For neutralization experiments, monoclonal neutralizing anti-human IFN-γ antibody (eBioscience, CA) was used to block IFN-γ in 1 × 10^5 ^aCD4S, while mouse IgG1 isotype was used as control. Antibodies were added to 1 × 10^5 ^aCD4S at 100 μg/ml and kept at room temperature at least 30 min, then was used to presensitize Hela cells for 2 days. The presensitized Hela cells were irradiated with 4 Gy, plated in 96-well plate at 5000 cells/well with 200 μl of fresh CM, and tested cell viability on day 5 using WST-1 reagent.

### Cell cycle analysis

Hela cells were plated in 24-well plate at 4 × 10^4^/well/0.8 ml fresh CM overnight, treated with IFN-γ, TNF-α, IFN-γ/TNF-α and 1 × 10^5 ^aCD4S for 48 hrs, followed by 4 Gy γ-irradiation. The irradiated tumor cells were plated in 24-well plate at 1 × 10^5^/well/ml of CM for 2 days. Apoptosis was measured by flow cytometry after tumor cells were labeled with PI and FITC-conjugated annexin-V. Flow cytometry was performed on a Becton/Dickenson FACStar II. Individual fluorescence intensity values of 20,000 cells were obtained for each sample.

### Western blot

Hela cells, resuspended in fresh CM at 5 × 10^5^/ml × 5 ml, were plated in T25 flask overnight, treated with 1 × 10^5 ^aCD4S for 48 h, followed 4 Gy of γ-irradiation, plated into 6-well plate at 2.5 × 10^5^cells/well with 3 ml of fresh CM for 2 days. The cells were washed twice in cold PBS and lysed in RIPA lysis buffer (Upstate Cell Signaling Solutions, NY) and protease inhibitor cocktail (Sigma-Aldrich, St. Louis, MO) and PMSF. After incubation on ice for 30 min, the lysates were centrifuged at 14,000 g at 4°C for 2 min. Protein concentration was determined by the Pierce Protein BCA Assay (Pierce, Rockford, IL). For Western blot analysis, 25 μg of protein for each sample was separated by sodium dodecyl sulfate-polyacrylamide gel electrophoresis and blotted onto a Hybond ECL nitrocellulose membrane (Amersham Pharmacia Biotech, Freiburg, Germany). Membranes were blocked for 1 h in TBST (Tris-HCl 20 mM, pH 7.5; NaCl 138 mM; Tween-20 0.1%) containing 5% non-fat milk at room temperature, probed with primary antibody for 1 h, washed three times with TBST, probed again with horseradish peroxidase-conjugated secondary antibody for 45 min, and washed again three times in TBST. Antigen-antibody reaction was revealed using enhanced chemiluminescence (ECL) procedures according to the manufacturer's instructions (Pierce, Rockford, IL). Bax antibody (Santa Cruz Biotechnology, Santa Cruz, CA) was used at 1:500 dilution. HRP-conjugated rabbit antimouse IgG (Cell Signaling, Beverly, MA) was used as a secondary antibody and was diluted at 1:3000.

### Statistical analysis

All data are presented as mean values with SD, unless indicated otherwise. The Student t-test was used to examine the statistical significance in the difference of growth assay data. Data were analyzed using the Medcalc software. P < 0.05 was considered statistically significant.

## Results

### Radiosensitizing effect of aCD4 on Hela cells

**Figure 1:**
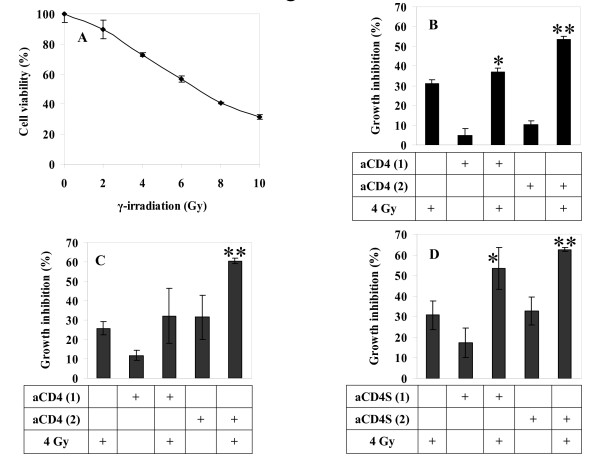
**Radiosensitizing effect of aCD4 on Hela cells**. (**A**) γ-irradiation dose response curve. Hela cells were treated with various doses of γ-irradiation, plated at 5 × 10^3^/well in 96-well plate containing 200 μl CM and cell viability was determined on day 5. (**B, C, D**) Suboptimal radiation dose of 4 Gy was chosen for subsequent experiments with aCD4 presensitization. On day -2, Hela cells were pre-sensitized with 1 × 10^4 ^aCD4 [aCD4 (1)] or 2 × 10^4 ^aCD4 [aCD4 (2)]. The cells were co-cultured directly with aCD4 (**B**), or via transwell system (**C**), or treated with conditioned medium (aCD4S) (**D**), followed (or not) on day 1 by 4 Gy γ-irradiation. Cell growth inhibition was determined on day 5 by WST-1 assay. All experiments were performed in triplicate and repeated at least 2 times. Data represent mean values of each triplicate ± S.D. Groups labeled ** (P < 0.01) were compared to irradiation alone group.

γ-radiation dose-response curves were generated for each tumor cell line. The cervical carcinoma cell line Hela was used as our tumor model (Fig. 1A). Based on this data, a suboptimal γ-radiation dose of 4 Gy, which yielded 27.3% growth inhibition, was selected for use in aCD4 presensitization experiments.

To investigate whether aCD4 could sensitize Hela cells to enhance the effect of γ-radiation, Hela cells were co-cultured with indicated doses of aCD4 for 2 days, then γ-irradiated with 4 Gy. Cell viability was determined using WST-1 assay on Day 5. Hela cell growth was inhibited by aCD4 in a dose dependent manner (Fig. 1B). Radiation alone resulted in 31.1 ± 2.1% cell growth inhibition. aCD4 alone, at a dose of 5 × 10^4 ^or 1 × 10^5^/ml, caused 4.8 ± 3.5% and 10.1 ± 1.9% cell growth inhibition, respectively; while presensitization with 5 × 10^4 ^or 1 × 10^5 ^aCD4/mL CM (hereafter called 5 × 10^4 ^or 1 × 10^5 ^aCD4), followed by γ-irradiation resulted in 37 ± 1.9% (P < 0.05 versus single agent, irradiation or aCD4 alone) and 53.3 ± 1.7% (P < 0.001 versus single agent) cell growth inhibition, respectively.

To investigate the contribution of cell-cell contact versus soluble factor(s) in the radiosensitization of tumor cells, a permeable transwell system was used to avoid the contact between tumor cells and aCD4. Similar degrees of growth inhibition were seen using the transwell system. In this system, 4 Gy irradiation alone, 5 × 10^4 ^aCD4 alone, or 1 × 10^5 ^aCD4 alone caused 25.8 ± 3.3%, 11.8 ± 2.6% and 31.5 ± 11.3% cell growth inhibition, respectively; while presensitization with 5 × 10^4 ^or 1 × 10^5 ^aCD4 via transwell, followed by γ-irradiation resulted in 32.2 ± 2.2% and 60.5 ± 1.3% cell growth inhibition, respectively. Compared with 4 Gy irradiation alone, a significant decrease in percentage of viable cells was seen in the 1 × 10^5 ^aCD4 plus irradiation group (P < 0.01) (Fig. 1C). To confirm that soluble factors were responsible for the observed activity, we examined the radiosensitizing effect of cell-free supernatants from aCD4 on Hela cells. Cell-free supernatants were collected from aCD4 after 48 h (hereafter called aCD4S). Hela cells were treated with aCD4S for 2 days, followed with 4 Gy γ-irradiation, and plated in a 96-well plate for cell viability assay on Day 5. Similar results were obtained as those in aCD4 and Hela cell-cell direct contact or in a transwell system. 30.8 ± 6.9%, 17.3 ± 7.1% and 32.9 ± 6.8% cell growth inhibition were seen in the 4 Gy irradiation alone, 5 × 10^4 ^or 1 × 10^5 ^aCD4S alone groups, respectively, while 53.4 ± 10.4% and 62.6 ± 1.0% cell growth inhibition were obtained when Hela cells were presensitized with 5 × 10^4 ^aCD4S or 1 × 10^5 ^aCD4S, respectively, followed by 4 Gy irradiation. Compared with 4 Gy of irradiation alone, a significant decrease in cell viability was seen in the combination of 5 × 10^4 ^or 1 × 10^5 ^aCD4S and irradiation groups (P < 0.05 and P < 0.01, respectively, versus single agent) (Fig. 1D). These data confirmed the role of aCD4, particularly soluble factors released from these cells, in sensitizing tumor cells to greatly augment the antitumor activity of γ-irradiation.

### aCD4 radio-sensitizing activity is irradiation dose dependent

**Figure 2:**
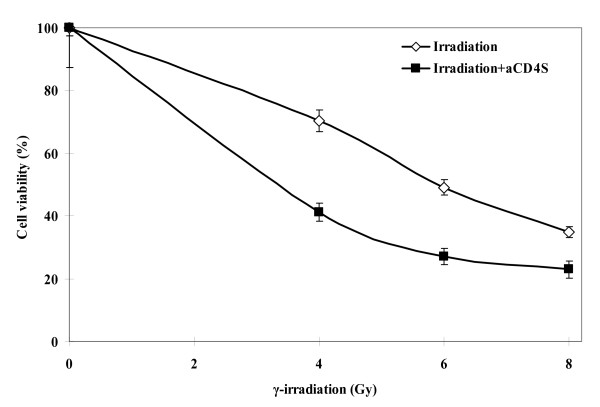
**aCD4 radio-sensitizing activity is irradiation dose dependent**. On day -2, Hela cells were pre-sensitized with or without conditioned medium (aCD4S) for 2 days then γ-irradiated on day 1 with various doses. The cells were plated at 5 × 10^3^/well in 200 μl in 96-well plate. Cell growth inhibition was determined on day 5 by WST-1 assay. All experiments were performed in triplicate and repeated at least 2 times. Data represent mean values of each triplicate ± S.D.

The data from Fig. 1 demonstrated the aCD4 dose dependent effect on the observed activity. Here we also demonstrated that the observed activity was also dependent on γ-irradiation doses. The survival fractions of Hela cells treated with 4 Gy, 6 Gy, or 8 Gy γ-irradiation alone were 88.51 ± 4.33%, 61.6 ± 5.9%, and 39.1 ± 4.1%, respectively; while those of Hela cells presensitized with 1 × 10^5 ^aCD4S, followed by the same doses of γ-irradiation were 53.8 ± 6.8%, 35.5 ± 5.5%, 26.1 ± 1.3%, respectively. The difference between irradiation alone and irradiation plus aCD4S was significant at each irradiation doses (P < 0.01) (Fig. 2).

### Confirmation of the radiosensitizing effect of aCD4 on the radioresistant glioma cell line LN229

**Figure 3:**
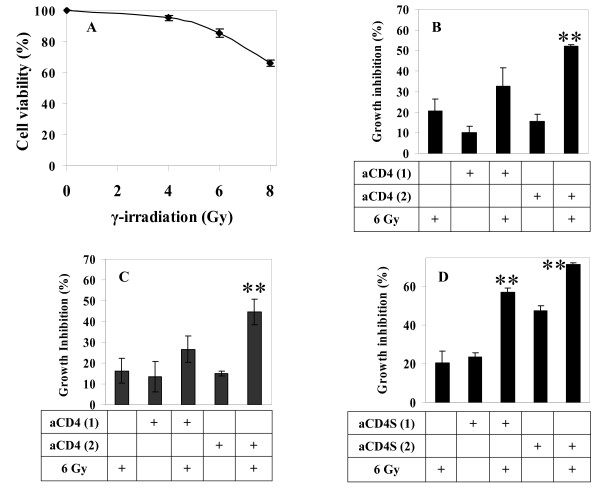
**Confirmation of the radiosensitizing effect of aCD4 on the radioresistant glioma cell line LN229**. (A) γ-irradiation dose response curve. LN229 cells were treated with various doses of γ-irradiation, plated at 5 × 10^3^/well in 200 μl CM in 96-well plate and cell viability was determined. (B, C, D) Suboptimal radiation dose of 6 Gy was chosen for subsequent experiments with aCD4 pre-sentization. On day -2, LN229 cells were pre-sensitized with 1 × 10^4 ^aCD4 [aCD4 (1)] or 2 × 10^4 ^aCD4 [aCD4 (2)]. The cells were co-cultured directly with aCD4 (**B**), or via transwell system (**C**), or treated with conditioned medium (aCD4S) (**D**), followed (or not) on day 1 by 6 Gy γ-irradiation. Cell growth inhibition was determined on day 5 by WST-1 assay. All experiments were performed in triplicate and repeated at least 2 times. Data represent mean values of each triplicate ± S.D. Groups labeled ** (P < 0.01) were compared to irradiation alone or  aCD4 alone.

To assess whether the radiosensitizing activity of aCD4 was unique to Hela cervical cancer cell line, similar experiments were carried out using the human glioma cell line LN229. Based on radiation dose response curve (Fig. 3A) and optimal presensitization condition for LN229, 6 Gy γ-irradiation was used instead of 4 Gy. This dose alone caused 14.6 ± 2.9% cell growth inhibition. LN229 cells were presensitized with aCD4 cells in a cell-cell direct contact manner, or via transwell system, or as cell-free conditioned medium (aCD4S) for 2 days, followed by 6 Gy γ-irradiation. After irradiation, the cells were cultured in CM containing 25% aCD4S for 5 days, and cell viability was measured using WST-1 reagent. In a cell-cell direct contact system, 6 Gy γ-irradiation alone, 5 × 10^4 ^aCD4 alone or 1 × 10^5 ^aCD4 alone caused 20.5 ± 6.0%, 10.0 ± 3.2% and 15.4 ± 3.4% cell growth inhibition, respectively; while presensitization with 5 × 10^4 ^or 1 × 10^5 ^aCD4, followed by 6 Gy γ-irradiation resulted in 32.7 ± 9.0% and 52.0 ± 0.8% cell growth inhibition, respectively. There was significant difference between the 1 × 10^5 ^aCD4 plus irradiation versus single agent, irradiation or aCD4 alone (P < 0.001 for both) (Fig. 3B). In the transwell system, 6 Gy γ-irradiation alone, 5×10^4 ^aCD4 alone or 1 × 10^5 ^aCD4 alone caused 16.3 ± 6.1%, 13.4 ± 7.2% and 14.9 ± 1.3% cell growth inhibition, respectively; while presensitization with 5 × 10^4 ^or 1 × 10^5 ^aCD4 via transwell, followed by 6 Gy γ-irradiation resulted in 26.7 ± 6.4% and 44.7 ± 6.7% cell growth inhibition, respectively. Compared single agent, irradiation or aCD4 alone, the 1 × 10^5 ^aCD4 plus irradiation groups significantly reduced LN229 cell viability (P < 0.01 for both) (Fig. 3C). In the aCD4S system, 6 Gy γ-irradiation alone, 5 × 10^4 ^aCD4S alone or 1 × 10^5 ^aCD4S alone caused 20.5 ± 6.0%, 23.4 ± 2.3% and 47.4 ± 2.9% cell growth inhibition, respectively; while presensitization with 5 × 10^4 ^or 1 × 10^5 ^aCD4S, followed by γ-irradiation resulted in 57.1 ± 2.3% and 71.7 ± 0.8% cell growth inhibition, respectively (P < 0.001 versus single agent for both) (Fig. 3D). The data demonstrated that aCD4, whether as whole cells cocultured directly with LN229 tumor cells (Fig. 3B) or via transwell membrane (Fig. 3C), or as cell free conditioned medium (aCD4S) (Fig. 3D), significantly enhanced the cell growth inhibition effect of γ-irradiation.

### Role of cytokines in the radiosensitization of tumor cells

**Figure 4:**
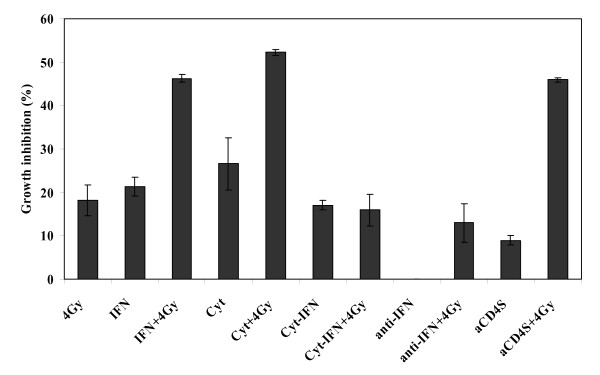
**Role of cytokines in the radiosensitization of tumor cells**. On day -2, Hela cells were pre-treated for 2 days with IFN-γ; combination of 10 cytokines, IL-2, IL-4, IL-6, IL-8, IL-10, IL-12p40, IL-13, IFN-γ, TNF-α and GM-CSF, (Cyt); combination of cytokines without IFN-γ (Cyt-IFN); aCD4S; or combination of cytokines in the presence of neutralizing anti-IFN-γ antibody. On day 1, the cells were treated (or not) with 4 Gy γ-irradiation. Cells were plated in 96-well plate at 5 × 10^3 ^cells/well in 200 μl CM and cell growth inhibition was determined on day 5 by WST-1 assay. All experiments were performed in triplicate and repeated at least 2 times. Data represent mean values of each triplicate ± S.D.

To understand the soluble factors involved in the observed activity, the aCD4 supernatants (aCD4S) were screened for the presence of 13 common Th1 and Th2 pro-inflammatory and anti-inflammatory cytokines as described in materials and methods, and were found to contain various concentrations of IL-2, IL-4, IL-6, IL-8, IL-10, IL-12p40, IL-13, IFN-γ, TNF-α and GM-CSF (data not shown). Presensitization of Hela cells with the combination of these cytokines, at concentrations equivalent to those in the supernatants of 1 × 10^5 ^aCD4S, significantly enhanced the growth inhibition effect of γ-irradiation (Fig. 4).

Of the 10 cytokines tested, only IFN-γ was found to significantly enhance the radiosensitivity of irradiation on Hela cells. We used IFN-γ-free cytokine combination as well as IFN-γ blockade experiments in the presensitization process to further confirm the essential role of IFN-γ in the observed activity. 4 Gy of γ-irradiation alone, IFN-γ alone, combination of all 10 cytokines previously described (Cyt), or 1 × 10^5 ^aCD4S alone caused 18.2 ± 3.5%, 21.4 ± 2.2%, 26.6 ± 6.1%, and 9.0 ± 1.1% cell growth inhibition, respectively. When Hela cells were presensitized with IFN-γ, Cyt, or aCD4S, followed by 4 Gy γ-irradiation, the cell growth inhibition was significantly increased to 46.3 ± 0.8% (P < 0.001 versus single agent), 52.3 ± 0.7% (P < 0.001 and P < 0.01 versus irradiation and aCD4S, respectively), and 45.9 ± 0.5% (P < 0.001 versus single agent).

In order to confirm the principal role of IFN-γ, it was omitted from Cyt (Cyt-IFN-γ), and the radiosensitization effect of the cytokine combination was lost, with 15.9 ± 3.6% cell growth inhibition observed versus 18.2 ± 3.5% for 4 Gy γ-irradiation alone (P > 0.05) (Fig. 4). To confirm our results, anti-IFN-γ monoclonal antibody (anti-IFN-γ) was used to block IFN-γ's activity in aCD4S, with IgG1 isotype antibody as control (Isotype). Treatment of Hela cells with 1 × 10^5 ^aCD4S pre-incubated with anti-IFN-γ, followed by 4 Gy γ-irradiation, abolished the radiosensitization effect of 1 × 10^5 ^aCD4S, resulting in 12.9 ± 4.4% cell growth inhibition versus 18.2 ± 3.5% for 4 Gy γ-irradiation alone (P > 0.05). The cell growth inhibition was 43.7 ± 0.5% for 1 × 10^5 ^aCD4S pre-incubated with Isotype plus 4 Gy γ-irradiation and 45.9 ± 0.5% for 1 × 10^5 ^aCD4S plus 4 Gy γ-irradiation (for both, P < 0.01 versus single agent alone) (Fig 4).

### Enhancement of radiosensitizing effect of IFN-γ by TNF-α

**Figure 5:**
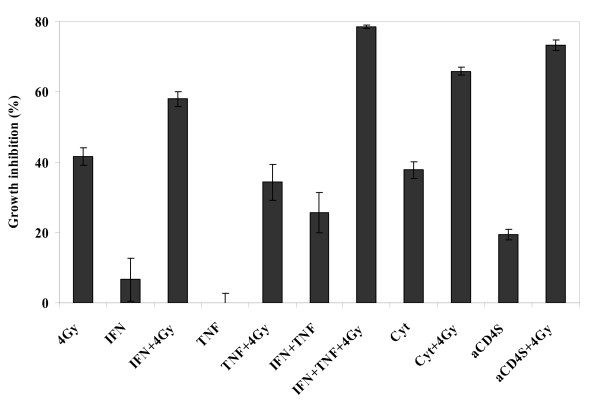
**Enhancement of radiosensitizing effect of IFN-γ by TNF-α**. On day -2, Hela cells were pre-sensitized for 2 days with IFN-γ, TNF-α, IFN-γ+TNF-α, combination of 10 cytokines (Cyt), or aCD4S, followed (or not) on day 1 with 4 Gy γ-irradiation. Cells were then plated in 96-well plate at 5 × 10^3 ^cells/well in 200 μl CM and cell growth inhibition was determined on day 5 by WST-1 assay. All experiments were performed in triplicate and repeated at least 2 times. Data represent mean values of each triplicate ± S.D.

For these experiments, 80 U/ml of IFN-γ and 5.6 ng/ml of TNF-α, concentrations equivalent to those in 1 × 10^5 ^aCD4S, were used. TNF-α by itself had no radiosensitizing activity. The treatment of Hela cells with TNF-α, followed by 4 Gy irradiation resulted in growth inhibition of 34.3 ± 5.1% vs. 41.6 ± 2.5% for irradiation alone (P > 0.05). In contrast, treatment with IFN-γ, followed by 4 Gy irradiation resulted in growth inhibition of 58.0 ± 2.1% (P < 0.01 versus irradiation or IFN-γ alone). Interestingly, when combined with IFN-γ, TNF-α significantly augmented the radiosensitizing effect of IFN-γ in Hela cells. Cell growth inhibition for IFN-γ + TNF-α followed by irradiation was 78.5 ± 0.6% versus 41.6 ± 2.5% for irradiation alone versus 25.8 ± 5.7% for IFN-γ + TNF-α alone (P < 0.001 for both). The difference between the IFN-γ + TNF-α followed by irradiation group and the IFN-γ followed by irradiation group was statistically significant (P < 0.001). The combination of all 10 cytokines as described previously (Cyt) and aCD4S were used as positive controls (P < 0.001 versus single agent treatment for both) (Fig. 5).

### Effect of aCD4 and cytokine presensitization on the redistribution of cell cycle phases and apoptosis of Hela cells

**Figure 6:**
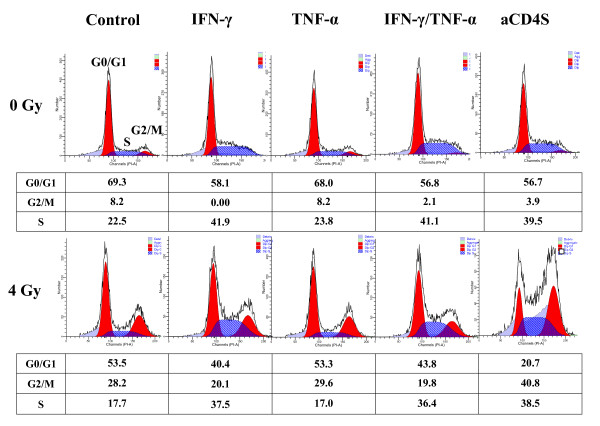
**Effect of aCD4 and cytokine presentization on the redistribution of cell cycle phases in Hela cells**. Representative flow cytometric profiles of Hela cells pre-treated for 2 days with IFN-γ, TNF-α, IFN-γ+TNF-α, or aCD4S, followed (or not) with 4 Gy γ-irradiation. The percentage of cells in G0/G1, G2/M or S-phase of the cell cycle is indicated under each curve. These experiments were repeated at least 2 times.

To investigate the mechanism underlying cell growth inhibition in our model, flow cytometric analysis was performed to assess the redistribution of cell cycle phases and apoptosis of Hela cells. Cell cycle analysis indicated IFN-γ, IFN-γ/TNF-α combination, or aCD4S shifted Hela cells into S-phase; while TNF-α alone had no effect, and TNF-α did not contribute to the activity seen with the IFN-γ/TNF-α combination. 4 Gy of γ-irradiation induced G2/M-phase arrest in Hela cells. While the presensitization of Hela cells with IFN-γ, TNF-α, or IFN-γ/TNF-α combination before the γ-irradiation did not affect γ-radiation induced G2/M-phase arrest, presensitization of Hela cells with aCD4S increased the γ-irrradiation-induced G2/M-phase arrest from 28.2% to 40.8% (Fig. 6).

**Figure 7:**
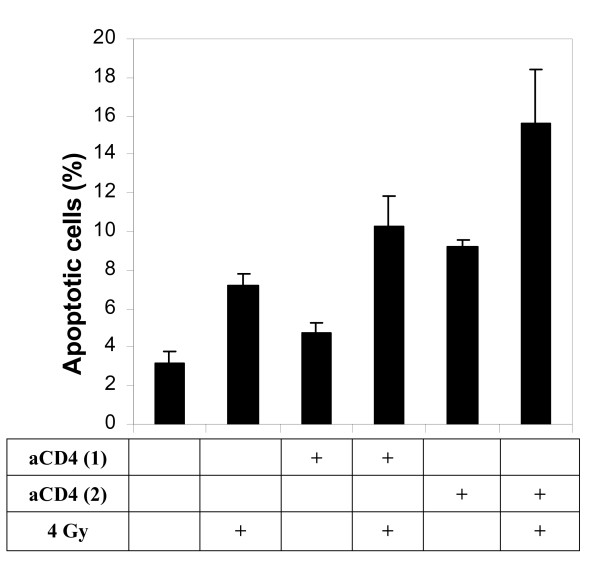
**Effect of aCD4 and cytokine presensitization on apoptosis in Hela cells**. Hela cells were pre-treated for 2 days with 1 × 10^4 ^aCD4 [aCD4 (1)] or 2 × 10^4 ^aCD4 [aCD4 (2)], followed (or not) by 4 Gy γ-irradiation. Tumor cells were labeled with FITC-conjugated annexin-V and PI and analyzed by flow cytometry. All experiments were repeated at least 2 times. Data represent mean values of each triplicate ± S.D.

Apoptosis analysis was performed two days after irradiation (as opposed to five days for cell viability assay). As shown in Fig. 7, the percentage of apoptosis was 7.15 ± 0.63% with 4 Gy γ-irradiation alone, 4.70 ± 0.57%, respectively, with 5 × 10^4 ^aCD4 alone, with 1 × 10^5 ^aCD4 alone, while the numbers increased to 10.30 ± 1.56% in 5 × 10^4 ^aCD4 plus 4 Gy of irradiation, and 15.65 ± 4.60% in 1 × 10^5 ^aCD4 plus irradiation. The differences were significant (p < 0.05) for 1 × 10^5 ^aCD4 plus 4 Gy group versus irradiation alone or aCD4 alone (Fig. 7).

### aCD4 presensitization followed by irradiation enhanced Bax expression

**Figure 8:**
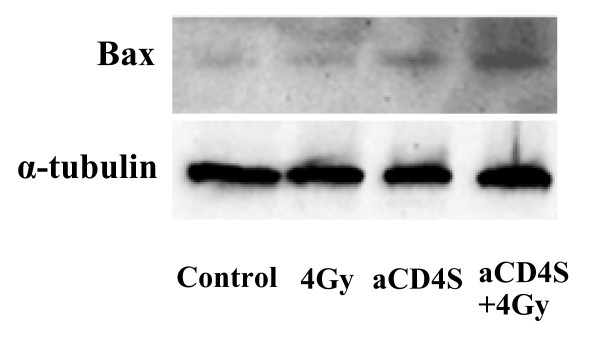
**aCD4S presensitization followed by irradiation enhanced Bax expression**. Hela cells were plated in T25 flask at 5 × 10^5^/well/5 ml CM overnight, treated with aCD4S for 2 days, followed by 4 Gy γ-irradiation, plated into 6-well plate at 2.5 × 10^5^cells/well with 3 ml CM for 2 additional days. Western blot analysis was performed to evaluate the expression of the pro-apoptotic protein Bax.

To further investigate the mechanisms underlying the radiosensitizing activity of aCD4, western blot analysis was performed to evaluate the expression of the pro-apoptotic protein Bax. There was a slight increase in Bax expression when Hela cells were treated with aCD4S alone, compared with no treatment or γ-irradiation alone groups. However, Bax expression was highly upregulated in Hela cells presensitized with aCD4S followed by γ-irradiation (Fig. 8).

### General Role of aCD4 or IFN-γ/TNF-α combination in the radio-sensitization of cancer cells

**Figure 9:**
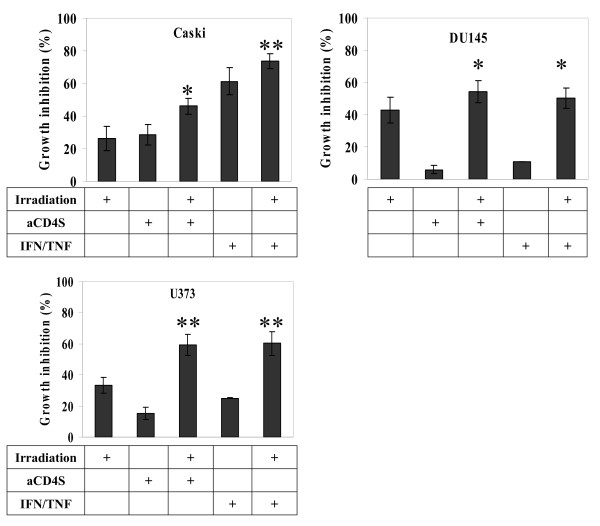
**General role of aCD4 or IFN-γ/TNF-α combination in the radio-sensitization of cancer cells**. Cancer cells from various histology were pre-treated with aCD4S or the combination of 160 U/ml of IFN-γ and 11.2 ng/ml of TNF-α were used to presensitize tumor cells for 3 days, followed by irradiation (4 Gy for a cervical cell line CaSki and prostate cancer cell line DU145, and 8 Gy for a glioma cell line U373). Similar results were seen in two independent experiments. All experiments were performed in triplicate and repeated at least 2 times. Data represent mean values of each triplicate ± S.D. Groups labeled * (p < 0.05) and ** (P < 0.01) were compared to irradiation alone group.

To assess the generality of aCD4 or IFN-γ/TNF-α combination as a radiosensitizer, we tested our approach on the cervical carcinoma cell line Caski, prostate carcinoma cell line DU145 and glioma cell line U373. Radiation dose response curves were generated for each cell line (data not shown). aCD4S or the combination of 160 U/ml of IFN-γ and 11.2 ng/ml of TNF-α were used to presensitize tumor cells for 48 hrs, followed by irradiation. Significant enhancement of cell growth inhibition was seen in each case (Fig. 9). In Caski cells, 4 Gy of γ-irradiation alone, 2 × 10^5 ^aCD4S alone or IFN-γ/TNF-α alone caused 26.2 ± 7.3%, 28.6 ± 6.3% and 61.3 ± 8.2% cell growth inhibition, respectively; while presensitization with 2 × 10^5 ^aCD4S or IFN-γ/TNF-α, followed by γ-irradiation resulted in 46.0 ± 4.7% (P < 0.05 versus irradiation alone) and 73.6 ± 4.4% (P < 0.001 versus irradiation alone) cell growth inhibition, respectively (Fig. 9A). In Du145 cells, 4 Gy of γ-irradiation alone, 2 × 10^5 ^aCD4S alone or IFN-γ/TNF-α alone caused 42.9 ± 7.9%, 6.0 ± 2.6% and 10.9 ± 0.1% cell growth inhibition, respectively; while presensitization with 2 × 10^5 ^aCD4S or IFN-γ/TNF-α, followed by γ-irradiation resulted in 54.1 ± 6.8% and 50.4 ± 6.3% cell growth inhibition, respectively. Compared with irradiation alone, aCD4S alone and IFN-γ/TNF-α alone treatments, aCD4S and IFN-γ/TNF-α combined with irradiation clearly inhibited the cell growth (P < 0.05) (Fig. 9B). In U373 cells, 8 Gy of γ-radiation alone, 2 × 10^5 ^aCD4S alone and IFN-γ/TNF-α alone caused 33.2 ± 5.2%, 15.2 ± 3.8% and 25.0 ± 0.3% cell growth inhibition, respectively; while presensitization with 2 × 10^5 ^aCD4S and IFN-γ/TNF-α, followed by γ-irradiation resulted in 59.3 ± 6.8% and 60.3 ± 7.5% cell growth inhibition. Compared with irradiation alone, aCD4S alone and IFN-γ/TNF-α alone treatments, aCD4S and IFN-γ/TNF-α combined with irradiation markedly inhibited the cell growth (P < 0.01 for both) (Fig. 9C). The results showed either aCD4S or the combination of IFN-γ/TNF-α could increase the radiosensitivity of these cancer cell lines to γ-irradiation.

## Discussion

We have demonstrated that pre-sensitization of tumor cells with non-specifically activated CD4^+ ^T cells greatly enhanced the apoptotic effect of γ-irradiation, and that soluble factors secreted from the activated CD4^+ ^T cells were primarily responsible for the observed effect. Of a number of Th1 and Th2 cytokines tested, IFN-γ seemed to play a major role in the radiosensitization of tumor cells. TNF-α, though inactive by itself, significantly augmented the radiosensitizing activity of IFN- γ. aCD4S, but not IFN-γ or IFN-γ/TNF-α combination, was found to enhance the γ-irradiation-induced G2/M phase arrest. In addition, Bax expression was highly upregulated in Hela cells presensitized with aCD4S followed by γ-irradiation. The radio-sensitizing activity of aCD4 is not uniquely observed in Hela cell line, but also in multiple other cell lines, including two glioma, one prostate, and another cervical cancer cell line.

Results from recent studies may also help provide other possible mechanisms that underlie the radio-sensitizing effect of aCD4. Inflammation and inflammatory cytokines were shown to generate nitric oxide (NO) through the induction of nitric oxide synthase, resulting in global inhibition of DNA repair activity in cholangiocarcinoma cells [23]. Other studies demonstrated that IFN-β or IL-24 down-regulated the DNA repair enzyme O6-methylguanine-DNA methyltransferase expression and activity in neuroblastoma and melanoma, respectively [24,25].

Interestingly, in our model while TNF-α had no direct cell inhibition effect either by itself or with radiation treatment, TNF-α was found to significantly enhance IFN-γ' radio-sensitizing activity when used in combination with IFN-γ. The reason for this effect is not clear. However, induction of NO and its subsequent release may be one of the underlying mechanisms. The addition of anti-TNF-α blocking antibody to murine macrophage cell lines prior to irradiation has been shown to inhibit NO induction by IFN-γ [26]. In addition, the combination of IL-1β, TNF-α, and IFN-γ, was shown to generate NO through the induction of NO synthesis [23]. NO has been shown to involve in DNA damage and inhibition of DNA repair [23,27].

Previous studies have shown that IFN-γ treatment reduced the expression of bcl-2 but did not alter that of Bax [28,29]. Our findings revealed that Bax levels were slightly increased in Hela cells with aCD4S treatment alone but substantially upregulated with aCD4S presensitization followed by γ-irradiation. Therefore, it is possible that the combination of bcl-2 reduction and Bax elevation, resulting in a greatly decreased bcl-2/Bax ratio, also plays a major role in tumor cell growth inhibition and enhanced apoptosis observed in our model.

Irradiation induced G2/M arrest in most cells [30]. Our data supported this finding. In addition, we have also shown that pre-treatment of tumor cells with conditioned supernatant from activated CD4^+ ^T cells (aCD4S) enhanced irradiation's effect on the cell cycle arrest in G2/M phase, which is also the cell cycle phase in which tumor cells are most sensitive to the DNA damaging effect from irradiation [31].

## Conclusion

Our finding showed that activated CD4^+ ^T cells enhanced radiation effect through the cooperation of interferon-γ and TNF-α. The study also suggest possible molecular and cellular mechanisms that may help explain the radio-sensitization effect of activated lymphocytes, and may provide an improved strategy in the treatment of cancer with radiotherapy.

## Abbreviations

aCD4: activated CD4+ T cells; aCD4S: conditioned supernatant from aCD4; CM: complete medium; PBMC: peripheral blood mononuclear cells.

## Competing interests

The authors declare that they have no competing interests.

## Authors' contributions

YW carried out most experiments and drafted the manuscript. SR isolated PBMC from healthy donors' blood. HK participated in the design of the study and helped to draft the manuscript. All authors read and approved the final manuscript.

## Pre-publication history

The pre-publication history for this paper can be accessed here:

http://www.biomedcentral.com/1471-2407/10/60/prepub
